# The liver in sepsis: molecular mechanism of liver failure and their potential for clinical translation

**DOI:** 10.1186/s10020-022-00510-8

**Published:** 2022-07-30

**Authors:** Dustin Beyer, Jessica Hoff, Oliver Sommerfeld, Alexander Zipprich, Nikolaus Gaßler, Adrian T. Press

**Affiliations:** 1 Department of Anesthesiology and Intensive Care Medicine, Jena University Hospital, Am Klinikum 1, 07747 Jena, Germany; 2 Center for Sepsis Control and Care, Jena University Hospital, Bachstr. 18, 07743 Jena, Germany; 3 Department of Internal Medicine IV, Jena University Hospital, Am Klinikum 1, 07747 Jena, Germany; 4 Pathology, Jena University Hospital, Am Klinikum 1, 07747 Jena, Germany; 5 Medical Faculty, Friedrich-Schiller-University Jena, Kastanienstr. 1, 07747 Jena, Germany

**Keywords:** Liver failure, Cholestasis, Intensive care, Sepsis, Inflammation, Molecular medicine, Personalized medicine

## Abstract

Liver failure is a life-threatening complication of infections restricting the host's response to infection. The pivotal role of the liver in metabolic, synthetic, and immunological pathways enforces limits the host's ability to control the immune response appropriately, making it vulnerable to ineffective pathogen resistance and tissue damage. Deregulated networks of liver diseases are gradually uncovered by high-throughput, single-cell resolved OMICS technologies visualizing an astonishing diversity of cell types and regulatory interaction driving tolerogenic signaling in health and inflammation in disease. Therefore, this review elucidates the effects of the dysregulated host response on the liver, consequences for the immune response, and possible avenues for personalized therapeutics.

## Background

Sepsis, a life-threatening organ dysfunction caused by a complex dysregulated host response to infection, is the leading cause of death in intensive care units worldwide (Liu et al. 2014; Woźnica et al. 2018; Singer et al. 2016). Rudd et al. estimations suggested 48.9 million sepsis cases globally in 2017 (Rudd et al. 2020). Organ failure is designated to discriminate a regulated from a dysregulated host response and is associated with high mortality (Schuler et al. 2018). In contrast to local infections, sepsis is a systemic and multifaceted alteration of the immunological function. The upregulation of both pro- and anti-inflammatory pathways leads to a massively increased release of chemokines and pathogen-related molecules. In the current understanding, the dysregulated host response to infection causes initial pro-inflammatory responses and inadequate anti-inflammatory responses that control pathogen clearance efficacy and limit tissue injury (Bauer et al. 2018). Early microangiopathies that activate complement and coagulation pathways deteriorate organ function. In addition, complex interactions of molecular mediators and microbial structures (pathogen-associated-molecular patterns—PAMPs) with Toll-like receptors (TLRs] on the surface of Antigen-presenting cells initiate the immunological signaling cascade. Due to these inflammatory alterations, a metabolic shift towards anaerobic metabolism and hyperglycemia (mainly forced by increasing insulin resistance) can be remarked. The combination of immunological, metabolic, microvascular, and coagulation alterations consequently leads to organ dysfunction, limiting patient outcomes (Font et al. 2020; Rubio et al. 2019). The liver in sepsis has become necessary for deciphering the complex sepsis pathophysiology. The liver controls the global immune function through various local immune cell populations that secrete immune-modulatory cytokines stimulating adaptive immunity. Furter liver injury affects the parenchymal cells. In particular, hepatocytes' downfall affects both the global metabolism and innate and adaptive immune function due to their ability to secrete pro- and anti-inflammatory proteins and sequester immune-controlling endogenous and exogenous alarmins.

Thus, early liver failure frequently triggers multiple organ dysfunction syndromes and is associated with a poor outcome (Kasper et al. 2020). The liver's importance for the immune response in critical illness originates in its vast metabolic and immune-modulatory function. In health, the liver acts as a second line of defense against pathogens after the gut's mucosa (Kubes and Jenne 2018).

Besides challenges concerning diagnosis, therapeutic interventions for septic organ dysfunction are scarce. Intensive care patient management focuses on adequate fluid restriction, stabilizing ventilation, and treating the infection (Gotts and Matthay 2016). A specific therapeutic approach to heal the liver is not available so far. In the case of liver dysfunction, organ replacement is challenging and demanding, and most bridges within short time intervals, allowing the liver to regenerate itself. Ultimately, liver transplantation is the last medical resort to revive a non-regenerating failed liver (Samuel and Coilly 2018). Unfortunately, patients who suffer from liver failure during sepsis are often present with long-term sequelae of liver dysfunction when the initial insult triggers inflammatory cascades leading to chronic liver failure (Wang et al. 2014). Thus, 27 to 48% of long-term mortality could be observed over the last decades, based on the worsening circumstances caused by cholestatic liver failure (Horvatits et al. 2019).

**Fig. 1:**
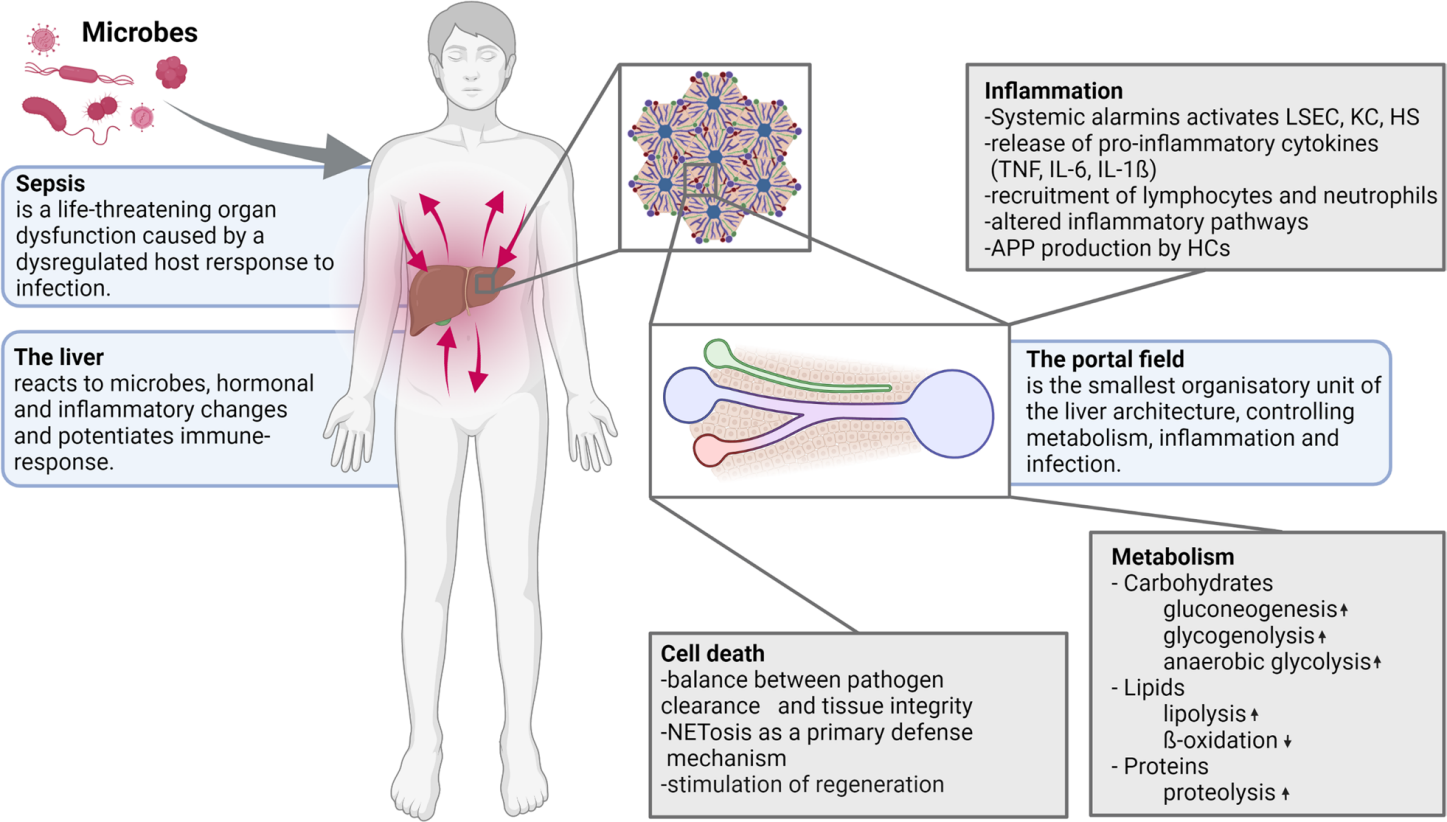
The liver in sepsis. Key changes in the liver tissue during systemic infection that ultimately result in a dysbalanced host response and liver failure. (LSEC - Liver sinusoidal endothelial cell; KC - Kupffer cell; HS - Hepatic stellate cell)

Infections cause an acute phase response, accompanied by metabolic and immunological changes in the liver (Sun et al. 2020). It has become apparent that the livers' response to infection depends on multiple factors, including genetic predisposition, gender, the current immunological status, pre-existing injuries, and the invading pathogen (Schaarschmidt et al. 2018). This complexity might be one reason why no specific treatment against septic liver failure has been discovered for its clinical use (Canabal and Kramer 2008). At the same time, preclinical studies demonstrate the great potential of personalized therapy of liver failure to improve patient care and sepsis outcomes (Rello et al. 2017; Itenov et al. 2018). This review summarizes critical pathophysiologic processes in septic liver failure and aims to demonstrate the opportunity for tailored treatment in septic liver failure (Fig. 1).

## Main text

### Key players in the liver during inflammation and infection

The liver is a significant immune modulatory organ where almost all immune and parenchymal cells comprise various immunological functions (Racanelli and Rehermann 2006). The liver acts as the second defense after the intestinal barrier, where microbes and their metabolites are conducted through the portal vein (Kubes and Jenne 2018; Racanelli and Rehermann 2006). The compilation of pro-and anti-inflammatory molecules and pathways is crucial to maintaining immunological homeostasis under physiological conditions. In the case of systemic infection, disturbances in inflammatory reactions consequently trigger a generalized immune response, possibly resulting in organ dysfunction that is coordinated by parenchymal, non-parenchymal, and infiltrating immune cells in the liver (Carranza-Trejo et al. 2021).

Hepatocytes, which account for 80% of liver parenchyma, are highly specialized and polarized endothelia-derived cells (Treyer and Müsch 2013). Hepatocytes finally play a pivotal role in inherent immune processes by counteracting bacterial invasion. Activated by pro-inflammatory cytokines, hepatocytes synthesize complement factors and various opsonins (Zhou et al. 2016). Besides, the regulation of iron homeostasis by hepcidin, transferrin, and hemopexin are beneficial in preventing bacterial growth (Zhou et al. 2016; Liu et al. 2021).

The liver sinusoidal endothelial cell (LSEC) is a specialized endothelial cell lining the capillaries of the liver, termed sinusoids. LSECs form a layer of epithelium with specialized fenestration termed sieve plates, allowing the exchange of various molecules through this barrier (DeLeve and Maretti-Mira 2017). LSECs also act directly as immune modulators by endocytosis and leukocyte recruitment. (Caparrós et al. 2020) The expression of scavenger receptors (SR] and Macrophage mannose receptors (MMRs) forms the molecular basis of clathrin-mediated endocytosis.

Another cell type around the liver sinusoid is the Kupffer cell (KC), a specialized and sessile macrophage (Bilzer et al. 2006). They represent most liver's myeloid cell population and guarantee a broad immunological function. Primarily endocytosis of foreign pathogens is the primary key of KCs, followed by antigen presentation and activation of other leukocytes with cytokine release (Sato et al. 2016). In addition, pattern recognition receptors (PRRs) like Toll-like receptors, complement receptors, and Fc receptors are ubiquitously expressed on the surface of these myeloid liver cells (Løvdal et al. 2000; Dixon et al. 2013; Faure-Dupuy et al. 2018). Cholangiocytes, in contrast, line the bile duct and represent the predominant cell type within the biliary tract. Accordingly, cholangiocytes are the first barrier to contact pathogens emerging from the bile, the duodenum. Thus, various PPRs are expressed on their surface, triggering intracellular signaling cascades (Chen et al. 2008). Yokoyama et al. demonstrated that cholangiocytes release IL-6 and IL-8 due to LPS stimulation, encouraging the hypothesis that cholangiocytes are involved in the innate and adaptive immune response during sepsis (Yokoyama et al. 2006). The hepatic stellate cells (HSCs), also known as Ito cells, typify another perisinusoidal cell inside the liver architecture. After activation, they further deposit collagen, the most prominent extracellular matrix protein in the liver, and inherit a vast metabolic function by regulating vitamin A storage and circulation (Friedman 2008). As pericytes, these cells are predominantly situated in the space of Disse, acting as liver resident antigen-presenting cells to activate natural killer T cells (NKT cells) through lipid antigens. Various hepatic cytokines and metabolites modulate the HSC activation and triggering. Secretions of IL-4 and IL-13 synthesized by CD8^+^ T and NKT cells as well as IL-17 and TNF-$\alpha$ from dendritic cells, macrophages, and Th17 T cells are crucial molecules, which activate HSCs (Puche et al. 2013). In contrast, TRAIL, IFN-$\gamma$, IL-22, and IL-10 counteract the pro-inflammatory response (Kinnman et al. 2003).

Mucosal-associated invariant T (MAIT) cells, a subpopulation of "innate-like" T cells, recognize preserved antigens from microbial riboflavin synthesis (Dusseaux et al. 2011). They mainly maintain the hepatic immune surveillance system via responding to both inflammatory cytokines and PAMPs (Kurioka et al. 2016). Thus, MAIT cells represent an essential T-cell subset within hepatic tissue to ensure the immunological function in case of sepsis.

Neutrophils, of course, are the predominant population of granulocytes involved in various processes. Activation can be either induced directly via bacterial compounds or via the release of pro-inflammatory cytokines and complement factor C5a. Granules within the cytoplasm contain multiple antimicrobial molecules, e.g., lysozymes, defenses, serin-proteases, and hydrolases. Another strategy of neutrophils to fight against invasive pathogens is neutrophil extracellular traps (NET). The release of DNA during cell death promotes the formation of a network, holding histones and proteases to capture and kill bacteria.

### Molecular basis

#### The liver drives inflammation in sepsis.

The immunological function of the liver is carried out by various highly specialized immune cells and hepatocytes, which form a complex cellular network modulating a systemic immune response towards pathogens, resulting in tolerance and anti-inflammatory or pro-inflammatory reaction coordinated through cytokines (Table 1) (Trefts et al. 2017). The liver is connected to the hepatic artery and portal vein and reacts particularly potent to pathogens and infections that reach the bloodstream. The liver acts here not as a trigger for the systemic host response to blood-borne pathogens but as a filter, capable of eliminating pathogens, pathogen-associated molecular patterns, debris, cytokines, and other pro-inflammatory metabolites. Thus, the liver exerts an astonishing immunoregulatory capacity that has been proven critical for the sepsis outcome (Kubes and Jenne 2018; Bauer et al. 2013; Yan et al. 2014; Strnad et al. 2017).

**Table 1:** Major cytokines in the liver during sepsis, their primary local sources, and effects

Cytokine	Effect	Source	References
IL-1β	– Synergistic effects with TNF-α	KC, M	(Shen et al. 2020)
IL-6	– Stimulation of acute-phase reaction	HC, KC, LSEC	(Schmidt-Arras and Rose-John 2016; Kawasaki et al. 2010)
IL-8	– Activation of neutrophils	HC	(Rajarathnam et al. 1994)
TNF-α	– Stimulation of IL-6 synthesis – Cell death induction	KC, N, CA	(Kishimoto 2010; Zhao et al. 2020)
IFN-${\varvec{\gamma}}$	– HC apoptosis – Upregulation of TNF-α, CD14	NKT	(Horras et al. 2011; He and Sun 2018)
TGF-ß	– Triggers immunosuppression – Induces IL-6 release by LSECs	HSC, Platelet	(Zhou et al. 2020; Balaphas et al. 2020)
IL-10	– Downregulation of TNF-α – Inhibition of monocyte differentiation	HC, M	Choi et al. 2019; Wang et al. 2018)

*KC* Kupffer cell, *HC* hepatocyte, *N* neutrophil granulocyte, *CA* cholangiocyte, *NKT* natural killer cell, *LSEC* liver sinusoidal endothelial cell, *HSC* hepatic stellate cell, *M* Macrophage

The liver detects infections within its vast sinusoidal network lined by LSECs that house the KCs, HSCs, and lymphocytes. LSECs act as sentinel and antigen-presenting cells after endocytosis, PAMPs, and other debris stimulate and recruit lymphocytes of the adaptive immune system (Protzer et al. 2012; Mehrfeld et al. 2018). Therefore, they express various immune receptors, co-stimulators, pathogen recognition receptors (PRRs], and adhesion molecules. (Pandey et al. 2020) Furthermore, with their large surface and capacity to filter PAMPs and pathogens, they shield Kupffer cells and hepatocytes from significant exposure to those xenobiotics. They would excessively recruit neutrophils situated in sinusoids that otherwise would react towards the invading pathogens (Haan et al. 2020). Other neutrophils in the liver had been depicted to undergo NETosis, a cell death mechanism in which the nuclear DNA is released during the cell death to form neutrophil extracellular traps (NET) holding histones and proteases, likewise a spider web, which can capture and damage or even kill bacteria (Denning et al. 2019). Through phagocytosis, protease-containing granules, and NETs, the hepatic microcirculation might be constrained, resulting in localized ischemic invents potentiating injury and recruitment of neutrophils to cope with the increased amount of cellular debris (Denning et al. 2019; Chen et al. 2021). A vicious circle establishes if the system fails to compensate for the increasing infiltrating immune cells and subsequent tissue damage. This pathophysiological chain of events may be one of the most significant confounders for long-term fibrotic and cirrhotic sepsis sequelae (Tang et al. 2021).

Liver-resident macrophages are another local pathogen sensor: Kupffer cells and monocyte-derived macrophages directly kill circulating bacteria after engulfing them through phagocytosis, significantly improving pro-inflammatory cascades (Slevin et al. 2020). For the recognition and phagocytosis of pathogens, Kupffer cells express high concentrations of scavenger receptors, Toll-like receptors, complement, and antibody Fc receptors (Bennett et al. 2020).

In addition, the Activation of Kupffer cells results in the expression and secretion of various pro-inflammatory, chemoattractant cytokines, including TNF-$\alpha$, IL-6, and IL-1 $\beta$ (Wen et al. 2020). However, the secretion of Kupffer cell-derived cytokines also modulates the hepatic metabolic function (Metlakunta et al. 2017). Hepatocytes respond to the inflammatory stimulation through Kupffer cell-derived cytokines, particularly IL6 (Su et al. 2018), by decreasing the iron mobilization into the blood to reduce bacterial nutritional sources. Further, hepatocytes start to secrete acute-phase proteins (APPs) such as C-reactive protein (CRP), which can rise from nearly not detectable plasma levels to 400 mg dL^−1^ within a few hours upon inflammatory and infectious triggers. CRP and other acute-phase proteins are central regulators of the antimicrobial response by opsonising4 pathogens for macrophages and potentiating immune signals. However, the massive synthesis of APPs requires many metabolic resources. Consequently, hepatocyte housekeeping proteins, such as serum albumin synthesis and bile formation, are significantly reduced (Gulhar et al. 2018; Ehlting et al. 2021).

The various interactions between parenchymal and non-parenchymal cells with other immune cells, cytokines, APPs, and other humoral factors determine the inflammatory processes in the liver and distant organs. However, this intercellular network reaches a complexity that has not been wholly conceptualized until today.

The importance of hepatocytes as a non-specialized immune cell with no phagocytic activity for immune control is astonishing. Hepatocytes in the liver serve as bacterial scavengers, detoxifying cells for all xenobiotics, the primary source for APPs, and producing inflammatory cytokines (Protzer et al. 2012; Cardoso et al. 2021). All those effects modulate the systemic immune response. However, the price of liver damage due to the overwhelming inflammation and vicious circles between inflammation, immune cell recruitment, and liver cell death has to be paid frequently (Kubes and Jenne 2018; Robinson et al. 2016; Zheng and Tian 2019). Liver damage often aggravates hypoxic events, which are initially triggered by cardiac, circulatory (Xanthopoulos et al. 2019), or respiratory failure (Herrero et al. 2020), and NETs primarily meant to capture and defeat pathogens can result in micro-thrombus formation resulting in reduced sinusoidal perfusion and detrimental hemodynamic alteration (Bonaventura et al. 2021). The inflammatory signaling in LSECs includes the induction of iNOS (Wang and Peng 2021), which decreases a vasodilatory response and increases the secretion of ET-1 acting on stellate cells (Kwok et al. 2009; Brewster et al. 2020), which contract and torch the capillaries, restricting blood flow additionally.

On the level of hepatocytes, the internalization and downregulation of biliary transporters that eliminate bile acids, bilirubin, drugs, and other xenobiotics through the hepatobiliary route have become a hallmark of sepsis, resulting in cholestasis (Recknagel et al. 2012). Furthermore, even cholangiocytes themselves release in such a situation pro-inflammatory cytokine, predominantly TNF-$\alpha$ and IFN-$\gamma$ (Pinto et al. 2018). This expansion of the periductal inflammation further impedes chloride and bicarbonate secretion by cholangiocytes and, due to the resulting loss of otherwise passively excreted water, restricts bile flow.

In summary, a complex immune function, local and often diffuse hypoxic events, metabolic reprogramming of hepatocytes, ductal inflammation, cholestasis, and liver dysfunction is the cause and target of a dysregulated host response distinguishing sepsis from sepsis uncomplicated systemic infections.

#### Liver metabolism in life-threatening infection

The liver plays a central role in protein biosynthesis, glucose, and fat metabolism. Jaundice (disturbed secretion function), coagulation disorder (aggravated biosynthesis), and hepatic encephalopathy (disturbed metabolization of ammonia) characterize the clinical manifestation of acute liver injury (Koch et al. 2017). Under physiological circumstances, bile acids, bile salts, and xenobiotics are taken up into hepatocytes by basolateral (portal venous) transmembrane transporters (Phase 0 biotransformation) (Boyer 2013). Essential transmembrane transporter systems for the uptake of endo- and xenobiotics from the sinusoids are Organic Cation Transporter (OCT), Organic Anion Transporter (OAT), OAT Pumps (OATP), and the NTCP. On the canalicular side, inflammation reduces the anchoring of transmembrane transporters like BSEP and MRP2 (Recknagel et al. 2012). Above all, many drugs (for BSEP circa 600 substances) act as competitive inhibitors and impair the functionality of these transporters, thus triggering disturbances in the metabolism of endogenous or exogenous substances (Morgan et al. 2013). The direct interaction of several therapeutics (e.g., immunosuppressive-, antibiotic-, antifungal-, antidepressant- or antiepileptic drugs) on the biotransformation has to be addressed, whereby the additional damage to hepatocytes through inflammation processes can lead to the accumulation of substances impairing the elimination of the noxious substances (Shen et al. 2020). In turn, clinicians constantly adjust doses or discontinue the therapeutic agents, giving more extensive clinical drug monitoring programs.

In infection driven-inflammation and related liver failure, Phosphoinositide 3-kinase-$\gamma$ (PI3K $\gamma$) signaling has been suggested as a pacemaker for intrahepatic excretory liver failure in mice and humans (Recknagel et al. 2012; Press et al. 2021). PI3K $\gamma$-knockout mice and mice treated with the PI3K $\gamma$ inhibitor AS605240 did not develop liver failure but suffered from immunological side effects (Press et al. 2021). PI3K $\gamma$ stimulates the phosphorylation and activation of protein kinase B (Akt), finally activating mTOR. The internalization of canalicular transport proteins, e.g., MRP2, constitutes a hallmark in the progression of cholestatic injuries. The internalization then accumulates bile acids and various xenobiotics, which may aggravate liver and systemic injury (Wang et al. 2019).

Recent findings suggest a complex signaling network, considering bile acids as signaling mediators instead of reducing them to their detergent function within the intestine. Hence, the bile acid taurocholate activates adenylate cyclase, increasing intracellular adenosine monophosphate (cAMP) concentration. Followed by cAMP-dependent guanine nucleotide exchange factor (Epac) signaling, which activates Ras-related protein 1 (Rap-1) and Mitogen-activated protein kinase kinase (also known as MAPKK or MEK) signaling (Yu et al. 2018). Consequently, liver kinase B1/AMP-activated protein kinase (LKB1-AMPK) activation results in phosphorylation of cytoplasmic linker protein 170 (CLIP-170), which is essential for the polymerization of microtubules to maintain intracellular trafficking. Thus, mentioned pathways mainly ensure the polarization of hepatocytes to fulfill guided intracellular trafficking. The apical domain consists of transport proteins like ABC transporters (e.g., ABCB11/BSEP), ensuring the elimination of endo- and xenobiotics across the canalicular membrane into the bile. Ras-related protein 11a (Rab11a) and myosin Vb-dependent endocytosis and recycling processes guide the transport of proteins into the membrane. Current studies suggest a direct interaction between LKB1-AMPK and Rab11a or indirect through Rab11a-Fip1 with constitutive AMPK phosphorylation sites, ensuring apical trafficking (Fu et al. 2011a, 2011b).

Further, cAMP-driven regulation of small GTPases as Ras homolog family member A (RhoA) and Ras-related protein 13 (Rab13) are crucial for junction forming and apical constriction by regulating myosin light chain (MLC) and altering intracellular ATP levels. (Marzesco et al. 2002).

Besides impaired biotransformation and excretion pathways, protein biosynthesis is disturbed during sepsis and systemic inflammation. The liver reduces the synthesis of various abundant plasma proteins, e.g., albumin, to cope with the increased necessity of acute-phase proteins. Further, the liver balances anabolic and catabolic pathways to adapt to changing environments (Robinson et al. 2016).

Lipid metabolism represents a significant part of hepatic metabolic function. Key features can be summarized in three distinct processes. The uptake of lipids, fatty acids, and de-novo lipogenesis is the first step, followed by lipid storage, triglyceride formation, and cholesterol synthesis. Finally, lipolysis, ß-oxidation, and the formation of low-density lipoproteins are crucial for metabolism (Jones 2016). Complex lipid structures like phospholipids and sphingolipids also undergo significant changes during sepsis. Thus, increased activity of acid sphingomyelinase leads to upregulation of ceramides and sphingomyelin degradation, promoting endothelial stress and microcirculatory dysfunction (Chung et al. 2016).

Peroxisome proliferator-activated receptor alpha (PPAR $\alpha$) is a crucial mediator in lipid metabolism, which coordinates lipolysis (Wyngene et al. 2020). The regulation of transcription, together with retinoid X receptor (RXR), binds to PPAR response elements (PPREs). During sepsis, decreased PPAR $\alpha$ levels enforce an excess of free fatty acids, leading to disturbed lipid metabolism and lipotoxicity due to alterations in ß-oxidation (Wyngene et al. 2020). Accumulating free fatty acids (FFA) drive lipotoxicity due to mitochondria damage and apoptosis induction. The accumulation of lipids results in steatosis, which further aggravates liver dysfunction and leads to long-term sequelae (Wasyluk and Zwolak 2021). Paumelle et al. further demonstrated that adequate PPAR $\alpha$ function is crucial for sepsis survival, although the underlying signaling mechanisms still have to be elucidated (Paumelle et al. 2019). The liver receptors alpha and beta (LXR-α and LXR-ß) are another class of transcription factors mainly involved in cholesterol and fatty acid metabolism. Activated by cholesterol-derived molecules (mainly oxidized natural lipids, e.g., 22(R)-hydroxycholesterol (22(R)-OHCh) and 27-hydroxycholesterol), LXRs modulate cholesterol synthesis and membrane composition by upregulating proteins like ATP binding cassette transporters and sterol regulatory element-binding proteins (SREBPs) (Ramón-Vázquez et al. 2019; Liebergall et al. 2020) Additionally, LXRs enhance the synthesis of pro-inflammatory mediators, which further aggravates disease severity (Souto et al. 2020).

Low cholesterol levels can often be detected in septic conditions of different entities. Reduced plasma cholesterol concentration and decreasing high-density (HDL-C) and low-density lipoprotein cholesterol (LDL-C) can be acknowledged, while the underlying pathology remains unclear. Various studies suggest that total cholesterol in patients can be used as a prognostic marker for sepsis outcomes, where low levels correlate with increased mortality (Hofmaenner et al. 2022). Changes in the metabolic system seem to affect multiple pathways of energy substrates. Mitochondrial dysfunction plays a crucial role in deteriorating liver disease, resulting in highly altered interferences of liver metabolism. Driven by immunological stimuli, the hepatocytes change their expression mode by synthesizing acute-phase proteins to further reinforce the body's defense mechanisms against infection (Robinson et al. 2016; Wasyluk and Zwolak 2021). Thus, energy failure is crucial for sepsis development. Glucose deregulation seems to be a key feature in metabolic failure (Weis et al. 2017). Impaired gluconeogenesis contributes to ketogenesis within the acute phase of infection. Evolving insulin resistance and hyperglycemia preserve glucose homeostasis end ensure appropriate immune function. Insulin resistance thus contributes to the redirection of glucose to cells that do not rely on insulin, e.g., neurons and leukocytes, to ensure appropriate function (Wyngene et al. 2018). The development of insulin resistance is vast and based mainly on the stimulation of sympathetic neurons, up-regulation of counter-regulatory hormones, and direct actions of pro-inflammatory cytokines (Wasyluk and Zwolak 2021). In case of hypoxia within liver tissue, HIF-1$\alpha$ is activated to enforce the expression of glycolysis related proteins, e.g., hexokinase, glucose-6phosphate dehydrogenase, and pyruvate dehydrogenase. This compensative mechanism, together with the pyruvate lack entering the tricarboxylic cycle, increases lactate production (Wasyluk and Zwolak 2021). Anaerobic pathways partially compensate for failing glycolysis to maintain energy levels for appropriate organ function (Wang et al. 2016). Altered glycogen metabolism and breakdown of energy production is another co-founder of systemic energy depletion. Increased lactate levels reflect the mode of anaerobic metabolism, which is widely used in clinical diagnostics (Vincent and Bakker 2021). Lercher et al. additionally show that type I interferons potentially harm liver key enzymes involved in the urea cycle. Hence, an altered arginine-to-ornithine ratio leads to impaired antiviral T-cell response (Nishio and Rehermann 2019). This clearly illustrates the tight cooperation of immunological and metabolic functions carried out by the liver.

#### Cell death in liver failure in sepsis

Cell death mechanisms in hepatocytes are multifactorial events resulting from intracellular alterations of the micro-milieu (Luedde et al. 2014). The death of liver cells is associated chiefly with immune response and the activation of other (immune) cells (e.g., Kupffer cells). Apoptotic T-cells and natural killer (NK) cells are abundant in the liver (Bertolino et al. 2007). T-cells show a phenotype characteristic for apoptosis, demonstrating that the liver is a specific site for trapping and destructing activated T-cells. At the end of an immune response, many T-cells are trapped in the liver without undergoing apoptosis but phenotypic changes. The presumed mechanism is that the liver sequesters cells from the circulation that are already undergoing apoptosis. At the time of T-cell arrival in the liver, the cells are moribund but not dead. This mechanism is called the graveyard hypothesis (Bertolino et al. 2007). Similar mechanisms are known for NKT cells in the liver. Pathological conditions and biological activators of the immune system increase the number of NKT cells in the liver, thereby playing a diverse role in controlling liver injury, fibrosis, or regeneration. One function is to selectively kill early activated HSCs by producing specific cytokines to reduce liver fibrosis (Gao and Radaeva 2012).

Cell death is the first transition step into an irreversible stage of liver failure. It regulates liver homeostasis by contributing to hepatocyte loss and growth balance. An imbalance due to enhanced death of hepatocytes is responsible for various human liver diseases and contributes to liver sequelae such as fibrosis and cirrhosis (He et al. 2017; Aizawa et al. 2020). Besides hepatocytes' chemical or mechanical death, apoptosis, ferroptosis, and necrosis are commonly studied cell death mechanisms. Hepatocellular death is present in almost all types of human liver disease and is used as a sensitive parameter for detecting acute and chronic liver disease of viral, toxic, metabolic, or autoimmune origin. Different ways of cell death such as apoptosis, necrosis, and necroptosis trigger specific cell death responses and promote liver disease progression through distinct mechanisms.

In sepsis, the metabolism of hepatocytes is modified toward the inflammatory response and cell death (Woźnica et al. 2018). Increasing evidence suggests that apoptotic cell death, besides other cell death mechanisms like necrosis, plays an essential role in sepsis and regulates the outcome by immune cell depletion that reduces the patient's ability to eradicate infections. In general, the cell death mechanism in the liver during sepsis depends on the cell type and stage of the disease. Recent results suggest that necrotic hepatic cell death is predominant in septic patients with liver dysfunction (Bantel and Schulze-Osthoff 2009).

Classification of cell death mechanisms induced by an external stimulus distinguishes between three regulated signaling pathways—necrosis, apoptosis, and pyroptosis. All three depend on receptor activation by an external death stimulus (Fink and Cookson 2005). In liver diseases and septic conditions, necrosis is a common finding, mainly followed by progressive liver fibrosis. The pattern and extent of the necrotic area in liver biopsies are important information during patients' clinical evaluation. The underlying design of the necrotic area gives essential clues to the underlying cause (Krishna 2017). The response mechanism during sepsis and liver diseases, triggered by the necroptotic production of pro-inflammatory mediators, is complex and not wholly understood (Pinheiro Da Silva and Nizet 2009).

Besides necrosis, apoptosis is a well-known feature of liver diseases caused by various factors (e.g., alcohol, viruses, fatty acids, bile acids) (Wang 2014). Also, apoptosis seems to be involved during septic conditions. Hofer and colleagues examine the importance of the clinical biomarker cytokeratin-18 (CK-18) for predicting clinical outcomes in sepsis. Caspases, critical apoptosis enzymes, cleaved the intermediate filament protein CK-18 (Bantel and Schulze-Osthoff 2009). The liver-specific bile acids act as an inducer for apoptosis and necrosis, dependent on the hydrophobicity and current concentration (Woolbright and Jaeschke 2019). Cholestasis blocks bile flow blockage whether the point of obstruction occurs extrahepatically or intrahepatically. Bile acids are a primary constituent of bile, and thus one of the primary outcomes is acute retention of bile acids in hepatocytes. Therefore, retention of bile acids is a primary cause of the associated liver injury during acute or chronic cholestasis. Despite this, a surge of papers in the last decade has reported a direct role of inflammation in the pathophysiology of cholestatic liver injury. Furthermore, the constituency of individual bile acids that make up a great pool and their conjugation status are intimately involved in their toxicity, which varies between species. Finally, the role of bile acids in drug-induced cholestatic liver injury remains an area of increasing interest (Woolbright and Jaeschke 2019). This increased bile acid concentration is characteristic of cholestatic conditions. It is well known that critically ill patients with cholestatic disorder also often develop sepsis as they are closely related. Both cholestasis and sepsis express pro-inflammatory cytokines that result in impaired bile secretion (Horvatits et al. 2017). sepsis-associated cholestasis is a clinical example of inflammation-induced liver disease (Geier et al. 2006).

The type of cell death execution, in particular necrosis or apoptosis, is a continuing debate (Fickert and Wagner 2017). It seems likely that low concentrations of bile acids induce apoptosis, and high concentrations induce necrosis (Perez and Briz 2009). Several studies have characterized the cellular and molecular mechanisms of hepatocyte injury caused by the retention of hydrophobic bile acids (BAs) in cholestatic diseases. BAs may disrupt cell membranes through their detergent action on lipid components and promote the generation of reactive oxygen species that, in turn, oxidatively modify lipids, proteins, and nucleic acids and eventually cause hepatocyte necrosis and apoptosis. Several pathways are involved in triggering hepatocyte apoptosis. Toxic BAs can directly activate hepatocyte death receptors and induce oxidative damage, causing mitochondrial dysfunction and endoplasmic reticulum stress. When these compounds are taken up and accumulate inside biliary cells, they can also cause apoptosis. Another form of regulated cell death is pyroptosis, an inflammasome and CASP-1-dependent form of necrosis that could be seen in macrophages, HSCs, and hepatocytes (Cookson and Brennan 2001; Wu et al. 2019). Activated CASP-1 leads to the cleavage of gasdermin D, a pyroptosis-inducing factor, through the production of IL-1 and IL-18. The release of IL-1 and IL-18 causes local and systemic inflammation in the cell, ultimately causing cell death (Man et al. 2017). In septic liver injury, pyroptosis in hepatocytes is considered a defense mechanism against intracellular bacterial infections that PAMPs and DAMPs recognize (Wu et al. 2019). There has been increasing interest in pyroptosis as a novel form of pro-inflammatory programmed cell death. The mechanism of pyroptosis is significantly different from other forms of cell death in its morphological and biochemical features. Pyroptosis is characterized by the activation of two different types of caspase enzymes and by the occurrence of a pro-inflammatory cytokine cascade and immune response. Pyroptosis participates in the immune defense mechanisms against intracellular bacterial infections (Wu et al. 2019).

In contrast, excessive pyroptosis conduces to the development of liver diseases. The exaggerated inflammatory response releases hepatic danger signals, enabling the activation of the inflammasome and other cell death mechanisms (Wu et al. 2019). These processes can cause and aggravate liver fibrosis, hepatitis, or septic shock (Zheng et al. 2021).

NETosis, in which, upon infection or injury, neutrophils release extracellular content, called NET, is another form of cell death in hepatocytes. NETs have been the subject of research in innate immunity since their first description more than a decade ago. Neutrophils are the first cells recruited at sites of inflammation, where they perform their specific functions, including the release of NETs, which consist of web-like structures composed of granule proteins bound to decondensed chromatin fibers. This process has aroused interest, contributing to understanding how pathogenic microorganisms are contained within an organism. Currently, there are growing reports of new molecules involved in the formation and release of NETs. The accumulation of hepatic neutrophils in liver disease is associated with increased release of NETs, suggesting that NETosis is a central mechanism in the aggravation of liver dysfunction (Bukong et al. 2018). In sepsis, infiltrated neutrophils produce ROS, nitric oxide synthase (iNOS), and NETs which contain harmful molecules inducing tissue inflammation and injury (Denning et al. 2019). NETs are a form of innate immune response to bind microorganisms and prevent them from spreading into the whole body (Brinkmann et al. 2004). This shows that NETs possess antimicrobial activities to capture virulence factors and damage extracellular microbes (Riyapa et al. 2012). Furthermore, recent evidence has implicated NETs as platelet and coagulation activity initiators during sepsis. Thereby neutrophils cast NETs through the circulation of different organs, where they participate in host defense and contribute to the development of end-organ damage (McDonald et al. 2017).

**Fig. 2:**
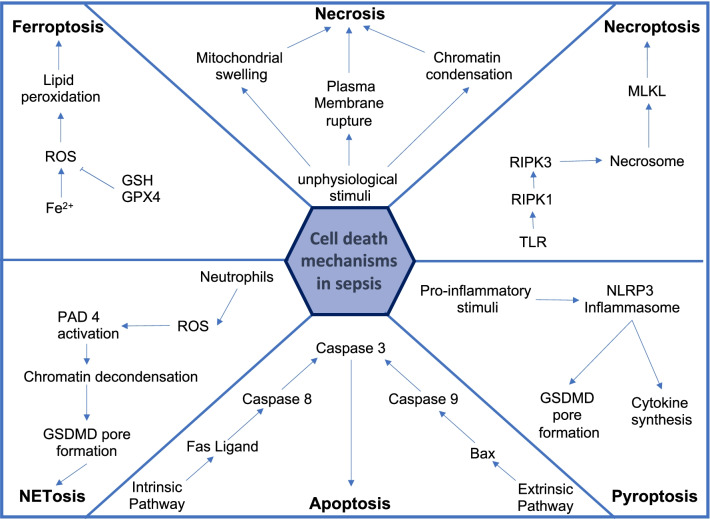
Main cell death mechanisms. The liver harbors parenchymal cells and different immune cell populations that contribute to immune reaction till their death through a regulated cell death mechanism. Hepatocytes are classical apoptotic cells since their death due to toxic metabolites occurs naturally and should not trigger inflammation. Recently, oxidative-regulated cell death Ferroptosis had been highlighted in various liver injuries, including sepsis driving reactive oxygen formation and inflammation. Necroptosis and pyroptosis are carried out by multiple cells after, e.g., TNF-$\alpha$ stimulation and drive inflammation during liver infection. NETosis dying neutrophils leave a vast number of extracellular debris and nucleotide nets to trap and destroy microorganisms even after their death. Finally, mechanically or chemical destruction of cells is apparent due to liver hypoxia or directly due to pathogen spread and toxic immune response

Ferroptosis represents a new entity, inducing cell death by increased non-transferrin-bound or catalytic iron, catalyzing ROS synthesis, leading to lipid peroxidation and cell damage (Dixon et al. 2012). Ferroptosis depends on intracellular iron and is morphologically, biochemically, and genetically distinct from apoptosis, necrosis, and autophagy. Ferroptosis may play a key role in liver diseases since various liver injuries can be associated with an increased iron level (Guyader et al. 2007; Lambrecht et al. 2011; Louandre et al. 2013). There are many indications that ROS and oxidative stress play an important role in initiating and progressing multiorgan dysfunction and injury in sepsis (Zhu et al. 2019). The most investigated ferroptosis regulator is the glutathione peroxidase 4 (GPX4), a lipid repair enzyme (Fig. 2). GPX4 catalyzes the oxidation of glutathione (GSH) to glutathione disulfide (GSSG), eliminating lipid peroxides and protecting the cell membrane against damage of polyunsaturated fatty acids. Therefore, the decreasing activity of GPX4 can induce ferroptosis. Studies show that reduced hepatic GPX4 expression correlates with increased ROS and iron levels, suggesting a potential role of ferroptosis for liver injury in sepsis (Wei et al. 2020). Additionally, ferroptosis has been identified as an essential cell death mechanism contributing to organ failure in the lung and heart (Li et al. 2020). Concluding these findings, ferroptosis remains one of the primary mechanisms aggravating multiorgan dysfunction. Elevated lipid peroxidation and catalytic iron levels strongly correlate with the mortality risk (Coillie et al. 2022).

### Therapeutic strategies

#### Anti-inflammatory therapy

Potential therapeutic aspects of reducing liver damage are anti-inflammatory modulation and preventive effects on liver functionality. Examples are the influence of PPAR agonists on lipid metabolism, the increase of bile flow, or the immunosuppressive effects of ursodeoxycholic acid (UDCA) (Koyama and Brenner 2017; Li et al. 2017). Studies further depict that FXR mediates pro-survival signals in hepatocytes by inhibiting the biosynthesis of bile acids, reducing the uptake of bile salts, and increasing their export through BSEP (Gomez-Ospina et al. 2016). In infection driven-inflammation and related liver failure, PI3K $\gamma$ signaling had been suggested to be a pacemaker for intrahepatic excretory liver failure in mice and humans (Recknagel et al. 2012; Press et al. 2021). PI3K $\gamma$ knockout mice and mice treated with the PI3K $\gamma$ inhibitor AS605240 did not develop liver failure but suffered from immunological side effects (Table 2). However, PI3K $\gamma$ knockout and AS605240 mice showed a neutrophil dysfunction associated with a cytokine storm that resulted in inadequate bacterial clearance and loss of any survival benefit (Recknagel et al. 2012; Press et al. 2021). Formulations resulting in an enrichment of AS605240 in hepatocytes reduced the immunological side effects without affecting the protective effects on liver function, ultimately increasing the survival rates of these mice (Press et al. 2021). While this approach seems promising, further studies are needed to validate these findings and translate such targeted therapies into clinical trials.

**Table 2:** Therapeutic strategies, their targets, and main actions to prevent liver failure

Drug	Study type		Target	Drug action in the liver	References
AS605240	preclinical	PCI mouse model	PI3K-γ	Biotransformation	(Press et al. 2021)
Dexmedeto-midine	preclinical	LPS rat model	TLR-4	TLR4/MyD88/NF-κB signaling downregulation	(Zi et al. 2019)
MCPIP1	preclinical	LPS mouse model	miR-9 SIRT-1	Decrease of Sirtuin1 in KC	(Zi et al. 2019)
Montelukast	preclinical	LPS rat model	Blockage of leukotriene receptor	Anti-inflammatory properties Lowered TNF-alpha levels Limitation of liver injury	(Donkers et al. 2019)
Pemafibrate	preclinical	CLP mouse model	PPAR-alpha	Reduction of accumulation of toxic lipid peroxidation products and cell death in liver	(Wyngene et al. 2020 Feb)
Resveratrol	preclinical	CLP rat model	SIRT-1	Inhibition of HMGB1 release	(González-Regueiro et al. 2020)
UAMC-3203	preclinical	CLP mouse model	Ferroptosis	Ferroptosis inhibition	(Coillie et al. 2022)
Vilobelimab	clinical	Multicenter, randomized, and placebo-controlled study	Neutralization of C5a	Decrease of IL-8, IL-10	(Bauer et al. 2021) (NCT02246595)
Wogonin	preclinical	LPS- and CLP mouse model	Nrf2	Anti-oxidative effects Inhibition of NF-κB-regulated pro-inflammatory signaling	(Dong et al. 2015)

There is also a potential benefit of blocking complement response by directly antagonizing C5a receptor 1 (C5AR1) (Sommerfeld et al. 2021). Investigations in a murine model of sepsis revealed evidence for improved outcomes in C5AR1 deficient mice by reduced pathogen load and mostly preserved liver function (Sommerfeld et al. 2021; Kusakabe et al. 2020). A current phase 2 trial with a novel monoclonal Anti-C5a antibody Vilobelimab promises beneficial effects on C5a neutralization. Thus, further investigations using C5a depletion are needed to evaluate the outcome in septic patients (Bauer et al. 2021).

Further experimental studies suggest the beneficial effect of different immunomodulatory drugs in preventing septic liver dysfunction.

Wogonin is a plant extract from *Scutellaria baicalensis*, which exerts its function by activating nuclear factor erythroid 2-related factor 2 (Nrf2), which indeed inhibits NF-$\kappa$ B guided pro-inflammatory signaling. The modulation of cytokine release enables a balanced immune response. Further improvements can be assigned by increased expression of antioxidant enzymes. The prevention of ROS formation prevents lipid oxidation and hepatocyte damage (Dong et al. 2015). Another strategy known in the therapy of bronchial asthma is the blockage of leukotriene receptors. According to the action of Wogonin, reduction of ROS formation and pro-inflammatory signals TNF-$\alpha$ ameliorates liver function in an LPS model of sepsis, assessed by liver enzymes (AST, ALT) and bilirubin (Donkers et al. 2019). Another pathway improving liver function is decreasing High-Mobility Group Protein Box 1 (HMGB1) translocation in hepatocytes by resveratrol administration. Increased sirtuin 1 (SIRT1) activity is fundamental for various processes regulating pro-inflammatory cytokines, metabolism, apoptosis, differentiation, and stress resistance. Resveratrol counteracts the septic downregulation of SIRT1 to prevent aggravating liver injury and preserve its function (González-Regueiro et al. 2020). Han et al. further developed the concept of SIRT1 upregulation to ameliorate septic outcomes. MCP-1 induced protein (MCPIP-1) is a protein involved in immunological signaling, conducting cytokine synthesis. Overexpression of MCPIP-1 in a murine model of sepsis leads to increased levels of SIRT1, providing the same efforts as reported for resveratrol. Thus, activators of MCPIP-1 are another new promising target to decrease septic liver failure (Zi et al. 2019). Dexmedetomidine reveals different effects in septic conditions. However, the main target is a subunit of the nicotinic acetylcholine receptor (α7nAChR). The activation inhibits the pro-inflammatory response, mainly driven by the TLR4 receptor, leading to NF-$\kappa$ B de-activation and decreased secretion of pro-inflammatory cytokines.

#### Metabolic therapy

Due to the critical and vast metabolic functions of the liver, targets involved in metabolism are broad and often involved in complex networks. Lipids are one of the significant moieties metabolized within the liver. Hence, the modulation of lipid metabolism appears to be a good attempt. Fibrates are activators of PPAR $\alpha$, which coordinates the breakdown of fatty acids. The administration of Pemafibrate reduced metabolic alterations during sepsis and prevented the aggravation of tissue damage (Wyngene et al. 2020). Tancevski et al. show that besides metabolic effects, fibrates ameliorate inflammation processes during sepsis due to neutrophil recruitment (Han et al. 2019).

However, BAs, such as ursodeoxycholic acid, have modulated BA-induced hepatocyte injury. The primary beneficial effects of treatment with ursodeoxycholic acid are protection against cytotoxicity due to more toxic BAs; the stimulation of hepatobiliary secretion; antioxidant activity, due in part to an enhancement in glutathione levels; and the inhibition of liver cell apoptosis. Other natural BAs or their derivatives, such as cholyl-N-methyl glycine or cholylsarcosine, have also aroused pharmacological interest owing to their protective properties (Perez and Briz 2009).

Another target might be the blockade of Sodium Taurocholate Cotransporting Peptide (NTCP) to reduce bile acid absorption, proapoptotic stimuli, and oxidative stress in hepatocytes (Li et al. 2017; Mohamadin et al. 2011; Xu et al. 2014). In addition to those direct protective benefits, liver functionality may also be positively affected indirectly by inhibiting immunomodulatory pathways.

#### Anti-liver cell death therapy

From a clinical perspective, the different cell death mechanisms are an excellent approach for new treatment strategies to maintain liver function in acute diseases and curb secondary inflammation in chronic diseases (Luedde et al. 2014). Clinical trials in patients with liver failure investigate various substances that influence apoptosis or necrosis.

Targeting ferroptosis appears to be beneficial in a subpopulation of MODS patients. The ferroptosis inhibitor UAMC-3203 increases survival in an experimental model of iron-overload induced organ dysfunction and in a genetically induced ferroptosis acute liver injury model. In addition, protection against multiorgan dysfunction and death makes the concept of ferroptosis inhibition a novel therapeutic strategy for preventing both single and multiorgan dysfunction (Coillie et al. 2022).

Clinical studies show that cell death mechanisms are a promising therapeutic approach for treating liver diseases. However, further research needs to elucidate the relationship between the different cell death mechanisms in hepatocytes to understand the pathological machinery of liver diseases (Dai et al. 2021). Concluding, the diversity of the described pathway in the context of cell death yields various targets for possible targeted therapies.

## Conclusions

The liver has a tremendous tolerogenic capacity maintained through a complex intercellular network of immune and parenchymal cells that provide sufficient anti-inflammatory signals to inflammatory signals constantly flooding in from the gut. Unfortunately, sepsis pro- and anti-inflammatory sepsis phases are dysregulated. Those reactions are recently classified into resistant and tolerogenic response patterns, balancing the host defense, i.e., pathogen killing, clearing, and organ injury. Disturbance of this balance results in a fragile state where pro-inflammatory signals can result in decompensation and life-threatening acute phase and liver dysfunction associated with poor prognosis. The liver's immunogenic, immunotolerogenic and metabolic capacities make the tissue and its cells pivotal in this novel concept. Recent therapeutic advances exert control over the hosts and liver's immune system and report tissue-protective and life-prolonging effects regardless of the pathogen clearance. Deciphering the molecular mechanism of organ failure and cellular communication in the liver now begins to stimulate the research of medicines enabling a targeted modulation of the liver's immune and inflammatory metabolic function to restore its tolerogenic signaling, regeneration, and metabolism, allowing it to break out of the vicious circle of multiple organ failure due to liver dysfunction.

## Data Availability

Data sharing does not apply to this article as no datasets were generated or analyzed during the current study.

## Abbreviations

Akt: Protein kinase B
ALT: Alanine transaminase
AMPK: AMP-activated protein kinase
APP: Acute phase protein
BA: Bile acid
BSEP: Bile salt export pump
C5AR1: Complemenet 5a receptor 1
cAMP: Cyclic adenosine monophosphate
CASP-1: Caspase 1
CCR2/5: C-C chemokine receptor type 2 and 5
CLIP-170: Cytoplasmic linker protein 170
CRP: C-reactive protein
DAMP: Damage-associated molecular pattern
Epac: Rap guanine nucleotide exchange factor 3
ET-1: Endothelin 1
FXR: Farnesoid X receptor
GPX4: Glutathione peroxidase 4
GSH: Glutathione
GSSG: Glutathione disulfide
HDL-C: High-density lipoprotein cholesterol
HIP/PAP: Hepatocarcinoma-intestine-pancreas/pancreatic associated protein
HSC: Hepatic stellate cell
IFN-$$\gamma$$: Interferon γ
IL-10: Interleukin 10
IL-13: Interleukin 13
IL-17: Interleukin 17
IL-1β: Interleukin 1β
IL-22: Interleukin 22
IL-4: Interleukin 4
IL-8: Interleukin 8
iNOS: Inducible nitric oxide synthase
KC: Kupffer cell
LDL-C: Low-density lipoprotein cholesterol
LSEC: Liver sinusoidal endothelial cell
LXR-ß: Liver X receptor ß
LXR-α: Liver X receptor α
MAIT: Mucosal associated invariant T cell
MAMP: Microbe-associated molecular pattern
MAPKK: Mitogen-activated protein kinase kinase
MLC: Myosin light chain
MMR: Macrophage mannose receptor
MRP2: Multidrug resistance-associated protein 2
mTOR: Mammalian target of rapamycin
NASH: Non-alcoholic steatohepatitis
NET: Neutrophil extracellular trap
NKT cell: Natural killer T cell
NTCP: Sodium taurocholate cotransporting peptide
OAT: Organic anion transporter
OATP: Organic anion transporter pumps
OCT: Organic cation transporter
PAMP: Pathogen-associated molecular pattern
PI3K: Phosphoinositide 3-kinase
PPAR α: Peroxisome proliferator-activated receptor alpha
PRR: Pattern recognition receptor
Rab11a: Ras-related protein 11a
Rab13: Ras-related protein 13
Rap-1: Ras-related protein 1
Reg3a: Regenerating islet-derived protein 3-alpha
ROS: Reactive oxygen species
SR: Scavenger receptor
SREBP: Sterol regulatory element-binding protein
TGF- β: Transforming growth factor β
TLR: Toll-like receptor
TNF-$\alpha$: Tumor necrosis factor α
TRAIL: Tumor necrosis factor related apoptosis-inducing ligand
UDCA: Ursodeoxycholic acid
VAP-1: Vascular adhesion protein 1
